# Improvement of the Solubility Amphotericin B Using Olive Oil Nanoemulsion Coated with Chitosan for More Effective Treatment of Zoonotic Cutaneous Leishmaniasis

**DOI:** 10.22037/ijpr.2021.115034.15162

**Published:** 2021

**Authors:** Elnaz Taghizadeh, Ali Khamesipour, Sepideh Khoee, Mahmoud Reza Jaafari, Seyed Abdollah Hosseini

**Affiliations:** a *Department of Chemistry, Alborz Campus, University of Tehran, Tehran, Iran. *; b *Center for Research and Training in Skin Diseases and Leprosy, Tehran University of Medical Sciences, Tehran, Iran. *; c *Polymer Laboratory, Department of Chemistry, College of Science, University of Tehran, Tehran, Iran. *; d *Nanotechnology Research Center, Biotechnology Research Center, School of Pharmacy, Mashhad University of Medical Sciences, Mashhad, Iran. *; e *Department of Medical Parasitology Mazandaran University of Medical Sciences (MAZUMS), Faculty of Medicine, Sari, Iran.*

**Keywords:** Amphotericin B, Olive oil, Nanoemulsion, Chitosan, Zoonotic cutaneous leishmaniasis

## Abstract

Amphotericin B (AMB) is a macrolide polyene antibiotic presenting potent anti-cutaneous *leishmania* activity. Nonetheless, its low water solubility, side effects, and toxicity have limited its therapeutic efficiency. The present study aimed to improve the solubility of AmB in oil-in-water using chitosan and determine its cytotoxicity on macrophages, as well as Leishmania major promastigote and amastigote. Olive oil, span 80, tween 80, AmB, and DMSO were employed as excipients, and nanoemulsions (NEs) were prepared by sonicator bath at 37 °C for 1 h at the highest power and stirred overnight with pH 5.5. Thereafter, chitosan was added to the NE and stirred overnight to obtain chitosan nanoemulsion (CNE). The CNE was optimized and investigated for different *in-vitro* parameters, such as droplet size, zeta potential, morphology, drug content, *in-vitro* drug release, and *in-vitro* cytotoxicity. Droplet size and zeta potential for CNE with AmB were obtained at 13.33 ± 1.3 nm, 30.90 ± 1.9 mV, respectively. Encapsulation efficiency and drug loading of the final CNE were reported as 100% and 0.64%, respectively. The results of in-vitro cytotoxicity revealed that CNE did not cause any cytotoxicity in macrophages. The CNE not only reduced drug toxicity for the macrophage but also had a suitable inhibition effect on the parasite. The CNE with AmB exerted an inhibitory effect on *L. major *promastigote/ amastigote phase. Furthermore, CNE with AmB represented a promising approach for leishmaniasis treatment. Therefore, the obtained outcomes of the IC_50_ proposed that the application of CNE can cause no toxicity and guarantees better quality drug release.

## Introduction

Leishmaniasis is an infectious disease caused by various *Leishmania* species. Based on clinical symptoms, leishmaniasis is divided into four groups, including cutaneous (CL), mucosal-cutaneous (MCL), visceral (VL), and diffuse-cutaneous (DCL) (9^1^-^3^). 

*Leishmania major* (*L. major*) is the causative agent of CL. In the intermediate host (sand-fly), this parasite multiplies in flagellate form or promastigote; however, in the final host (human and mammals), it is presented in un-flagellate form or amastigote. The amastigote infects macrophages, causing them to rupture and infect other macrophages (4-7).

Several chemical medications drugs are used to treat CL; nonetheless, the treatment of this disease is challenging, according to numerous reports of drug resistance. The second-line drugs for the treatment of this infection include Amphotericin B (AmB); however, its adverse side effects and poor drug-releasing have limited its therapeutic efficiency (6-8).

 The AmB is a polyene macrolide antibiotic produced by *Streptomyces nodosus*. It irretrievably binds to the sterols of membranes in the target cell and increases membrane permeability by making a cavity, resulting in cell death. The AmB is a second-line drug used to treat CL. Despite the high efficiency of AmB, its clinical application is restricted due to high hydrophobicity (9, 10). The formulation of Nanoemulsions(NEs)-loaded AmB reduces the side effects of AmB (11).

The NEs are colloidal scatterings of two immiscible solutions of oil and water. The NE is a very absorbing approach for transdermal drug delivery. The NEs mechanically release equally lipophilic and hydrophilic drugs via skin layers to achieve the blood current (11) They are thermodynamically unstable and need to use certain emulsifiers to stabilize the colloidal system on the oil-water interface. Tween 80 and Span 80 are known non-toxic ion-free emulsifiers widely used to coat nano-based drug delivery systems (12, 13). 

In their study, Mehrizi et al. hypothesized that all synthesized nano drugs were fully succeeded in recovering the *L. major*-related pathological effects in the infected footpad. Moreover, the findings of the mentioned study were demonstrated by real-time Polymerase chain reaction and revealed that Amphotericin B-chitosan and Betulinic acid nanochitosan worked well against *L. major* infection )^14^(. Chitosan is a herbal polysaccharide and a potentially biologically compatible material that is chemically versatile (-NH2 groups and various M(w)). These two basic properties are effective in drug delivery and tissue engineering. Therefore, the scientists have attempted to establish a plethora of formulations and scaffolds that demonstrated excessive usefulness in treatment modalities. The term “Chitosan” signifies a series of structurally varied chemical substances that may present various biodistribution, biodegradation, and toxicological effects (15).

Lipid-based formulations include a wide range of product requirements as follows: disease indication, route of administration, cost consideration, product stability, toxicity, and efficacy. All of these formulations are also commercially cost-effective for formulating the medicines which are administered via topical, oral, pulmonary, or parenteral routes (16). 

 Along the same lines, Cerqueira-Coutinho et al. developed an oil-in-water photoprotective and NE-containing chitosan. They performed preliminary studies aiming to select a surfactant to be used in this NE and assess the stability of the formulas by dynamic light scattering. The results of the stated study demonstrated that the synthesized photoprotective and antioxidant NE-containing chitosan was functional for more than six months (17).

 Biopolymers are generally used to increase the stability, rate of absorption and adjust the release load. Chitosan with a wide range of applications in different areas is also applied in the food industry and is considered appropriate for carrying lipophilic bioactive combinations(18, 19). The olive oil includes oleic acid in the oily phase, and it is efficient in increasing skin absorption (17).

In light of the aforementioned issues, the present study aimed to develop a nanoemulsion system of olive oil coated with chitosan as a natural positively charged polymer to increase the solubility of AmB and improve drug release to make it a more efficient and appropriate topical medicine for the treatment of CL. 

## Experimental

*Materials*

Amphotericin B (AmB), Surfactants Span® 80 and Tween® 80, Chitosan (28,510 kDa) with 90% deacetylation degree, PBS*** (***Phosphate-buffered saline), MTT (3-(4, 5-Dimethylthiazol-2-yl)-2, 5-diphenyltetrazolium bromide), FBS (Fetal Bovine Serum), and RPMI 1640 medium were purchased from Sigma-Aldrich, USA. Olive oil was kindly donated by Dr. Omid Rajabi from Dr. Rajabi Pharmaceutical Company, Mashhad, Iran. Mouse macrophage cell line J774A.1 (ECACC number 9051511) was purchased from Pasture Institute, Tehran, Iran.

*Preparation of NEs oil in water (o/w)*

Four samples were prepared with different ratios of surfactants was shown in Table 1, coded as S1 to S4, and used to determine the most stable one. Each NE was composed of various amounts of Span 80 and Tween 80 as surfactants. S3 was selected because of its stability (Span 80: 0.125 g, Tween80: 1.125 g, and HLB: 11.12) (9-10).

To prepare NEs through the oil-in-water technique, a solution of 1.125 g Tween 80 and 0.281 g olive oil as natural oil in 1000 µL deionized distilled water was vortexed at room temperature to produce a homogeneous phase. Then, 0.125 g Span 80 was added to the preparation and mixed vigorously. To prepare a clear oily NE, the mixture was incubated using a sonicator bath at 37 °C for 1 h at the highest power, followed by adding 5 mg of the prepared mixture oil to 10 mL deionized water, the NE stirred at 37 °C for 24 h and pH was adjusted to 5.5. Finally, the formulation was filtered using a 0.2 μm syringe filter and kept in the refrigerator until implementation.

*Preparation of AmB-loaded NEs*

**Figure 1:**
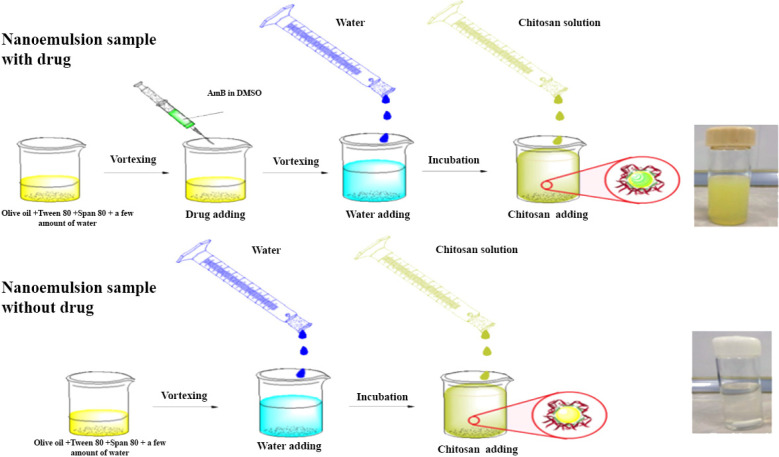
Schematic presentation of the CNE samples preparation loaded with and without AMB

To prepare AmB-loaded NEs, all the steps were the same as explained in section 2.2 accompanied by adding a solution of AmB (10 mg) in 5 mL Dimethyl sulfoxide (DMSO) to the oily phase before adding the oil phase to the deionized water (Figure 1) (15).

*Preparation of chitosan-coated NE (CNE)*

CNE was prepared by dissolving 10 mg of low molecular weight chitosan in 5 mL of 1% acetic acid, then 10 mL of sample S3 was added to the mixture, and the emulsion solution was stirred overnight; ph of the preparation was adjusted to 5.5 using NaOH. The formulated NE coated with chitosan was filtered using a 0.2 μm syringe filter and kept in the refrigerator until implementation. For the preparation of the control group, all the steps were repeated except AmB, which was not added into the second preparation (Figure 1).

*Drug release*

The freeze-dried CNE and NE (Alpha 1-2 Lo Plus, Germany) were each separately dissolved in 2 mL of PBS with pH 5.8 and put inside the dialysis bag molecular weight cutoff of 12,000 kD. Then, the bags were placed in 10 mL of PBS, spinning at room temperature for 196 h in pH 5.8. The release assessment was performed under constant stirring (100 rpm). Then about 10 mL of the PBS for each sample was taken and replaced with 10 mL of fresh PBS, then the absorption was read at 0.5, 1, 4, 6, 8, 21, 26, 27, 48, 119, 143, 167, and 191 h.

Samples were analyzed using UV-Vis Spectrophotometer (Cary 100 Bio, Varian, Australia) at a max of 406 nm. The release was calculated according to the following equation and shown in Figure 4.

Mt(n)=(vr × cn) + (vs ×∑ cm) 

Vr and Cn represent buffer volume outside the dialysis bag and concentrations, respectively. Also, Vs represents the removed volume, and ∑Cm is the sum of previous concentrations. 

Unlike CNE, no release was observed for NE, so the tests continued only with CNE, which revealed an appropriate release in 191 h.

*Calculation of encapsulation efficiency (EE) and drug loading (DL) of CNE loaded with AMB*

The amount of the drug in CNEs was calculated based on EE (%) and DL (%) using the following equations 

$\mathrm{EE}\left(\left(\frac{w}{w}\%\right)\right)\frac{\mathrm{weight of drug in nanoemulsion}}{\mathrm{weight of drug in feed}}$ × 100

$\mathrm{DL}\left(\left(\frac{w}{w}\%\right)\right)=\frac{\mathrm{weight of drug in nanoemulsion}}{\mathrm{weight of formulation components}}$ × 100

*Characterization of CNEs*

**Figure 2:**
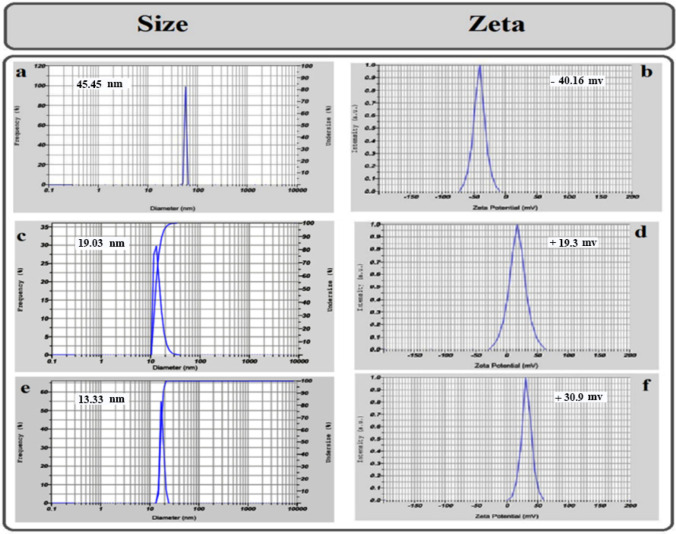
**(a) S**ize of NEs with AMB , (c) **S**ize of CNE without AMB , (e) **S**ize of CNE with AMB , (b) Zeta potential of NEs with AMB ,(d) Zeta potential of CNE without AMB , (f) Zeta potential of CNEs with AMB

The most stable formulation, NE with AMB, CNE with AmB, and CNE without AmB, were characterized by the dynamic light scattering (DLS) (Zeta Plus, Brookhaven, US), the average of hydrodynamic diameter, and surface charge (zeta potential) of NE with AmB, CNE with AmB and CNE without AmB were measured using DLS (Figure 2). 

**Figure 3:**
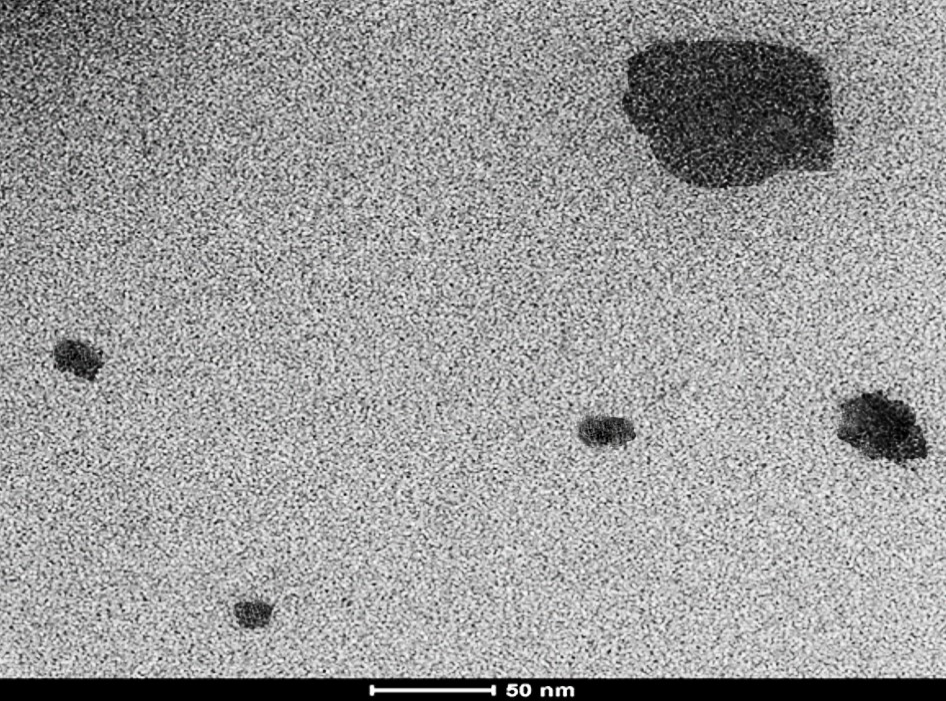
TEM images of AMB -loaded CNE.F

The morphology and size of CNE containing AmB were determined using a Transmission Electron Microscope (TEM). For this purpose, the CNEs were dispersed in distilled water, and a tiny amount of dispersion was transferred to a carbon-coated grid. Then, it was allowed to dry. The size was evaluated using HR-TEM (FEI TEC9G20, 200 kV) (Figure 3) (20).

*Macrophages culture*

The mouse macrophage cell line J774A.1 was cultured in RPMI-1640. MTT was used to check the effect of CNEs on cell-line growth. Briefly, 2×10^4^ cells/well were in each well and protected at 37 °C in a humidified atmosphere with 5% CO_2_. After 24 h, various concentrations of CNE loaded with AmB (0.025, 0.05, 0.1, 0.2 and 0.41 µg/mL) or CNE without AMB were added and incubated for an additional 24 h and 48 h, the plates were washed with PBS, and 10 μL of MTT was added to each well. After 4 h of incubation, the supernatants were removed, and 100 µL of DMSO was added to each well, and the optical density was read using an ELISA reader (GENS, Power wave xs2, Biotech, US) in 490 nm wavelengths. The experiment was conducted in triplicate. 

*Parasites culture*

*L. major* was isolated from BALB/c mice and cultured in a complete RPMI at 26 ± 1 °C.

*In-vitro effect of CNE on promastigote growth*

*L. major* promastigotes 5 × 10^5^ cells/well were added to each well, and then different concentrations of CNE with AmB and CNE without AmB (0.025, 0.05, 0.1, 0.2 and 0.4 µg/mL) were added to the wells and incubated at 26 ± 1 °C. MTT assay was performed at 24 h and 48 h.

*In-vitro activity against intracellular amastigotes*

J774 A.1 cell was cultured on the slides and incubated at 37 °C with 5% CO_2_ for 24 h (1 ×10 ^6^ cells/well). Then, *L. major *promastigotes harvested at stationary phase were added to each well at the promastigotes to J774 at a ratio of 10:1 and incubated for 24 h, then the coverslips were washed by PBS to remove extracellular promastigotes and different concentrations of CNE loaded with AmB and CNE without AmB were added to the wells and were incubated for an additional 24 h and 48 h at 37 °C and 5% CO_2_. At these time points, the coverslips were washed by PBS, fixed with methanol, and stained by Giemsa. The number of infected macrophages and amastigotes in 100 cells was determined by using a high-power microscope (B-383PL OPTIKA). The IC_50_ was calculated according to the following equation. 

Thepercentage of ingibition (%)=1-(the number of amastigote per 100 treated macrophages the number of amastigote per 100 untreated macrophages ) × 100

*Statistical analysis*

GraphPad Prism 7 (GraphPad Software Inc., USA) was used to generate and analyze the data. To evaluate the differences between the means, the one-way ANOVA with the t-test was applied. The p-value of less than 0.05 was considered statistically significant.

## Results

*Characterization of cationic nanoemulsion formulations*

The Zeta potential of cationic nanoemulsions (CNEs) was evaluated to determine their surface charge. The test was performed for three types of emulsions: 1) NE with AmB, 2) CNE without AmB, and 3) CNE with AmB. NE with AmB demonstrated negative charges, CNE without AmB displayed positive charges due to NH_2_ groups, and CNE with AmB showed more significant positive charges.

Size analysis illustrates that all NEs have a droplet size smaller than 50 nm. As the findings demonstrated, the size of CNE with AmB was fixed after 12 months. The size of CNE was smaller than NE due to the strong interaction between positively charged chitosan and negatively charged oily phase (Figure 2). The NE size was calculated at 45.45 nm with a zeta potential of -40.16 mV. After the addition of chitosan to the NE (CNE), the particle size was 19.03 nm, and the zeta potential was 19.3 mV which was smaller due to positively charged chitosan amine groups. Furthermore, the size of CNE with AmB was 13.33 nm, and zeta potential was 30.9 mV (Figure 2).

The transmission electron microscopy (TEM) investigation was carried out for the final sample without any exceeded agitation, and the results were compared with Dynamic Light Scattering (DLS) data (Figure 3). The outcomes illustrated that the TEM results for most particles were comparable with the size obtained by DLS measurement for CNE with AMB (Figure 3).

*In-vitro drug release*

The drug release, which occurred only for the drug-loaded CNE, did not illustrate any particular release for 200 h. It seems that the electrostatic interactions between drug and chitosan overcome the poor hydrophobic interactions between drug and oil in the core. Consequently, chitosan leads to drug transfer from the innermost position to the outer media. Therefore, CNE with AmB was selected to perform the rest of the experiments. Using the mentioned equations, Encapsulation efficiency (EE%) and drug loading capacity (DL%) of the final CNE were obtained at 100% and 0.64%, respectively.

*Cytotoxicity assay for macrophage and promastigote*

The results pointed out that the formulations induced no toxicity on J774 A.1, and the growth rate with the concentrations of 0.025, 0.05, 0.1, 0.2, and 0.41 µg/mL were similar to the AmB alone (Figure 5). No toxicity was observed for macrophages in any of the CNE with AMB, CNE without AmB, and AmB alone. The results of different concentration effects of CNE with AmB induced inhibition of growth of *L. major* promastigotes as presented in Figure 6. As depicted in Table 2, the IC_50_ calculation for AMB in 48 h demonstrated more effective results. In comparison with 24 h for CNE with AMB, a decrease in IC_50_ was observed in 48 h; however, it was not statistically significant.

*Effect of cationic nanoemulsion on amastigote*

The inhibition rate of amastigote was higher at 48 h in CNE with AmB. Different concentrations of CNE with AMB (0.05, 0.1, and 0.4 µg/mL) did not affect the macrophage growth, which might be due to the composition of chitosan, which is a natural polysaccharide. No toxicity was observed when CNE without AMB was used (Figure 7). As displayed in Table 3, the IC_50_ calculation for AmB in 48 h demonstrated more impressive results. Compared to 24 h for CNE with AmB, an increase was observed in IC_50_ in 48 h; however, it was not statistically significant. Furthermore, in the microscopic examination, CNE with AmB reduces the number of amastigotes in comparison with CNE without AmB.

## Discussion

The present study strived to dissolve AmB in the oil phase of a topical emulsion. The oil nanoemulsion was designed to have a positive charge and be more suitable for topical use; therefore, this operation was performed using chitosan polymer and in interaction with olive oil. In general, the reaction between olive oil and chitosan can be carried out in different ways.

The application of low-acetylation chitosan will result in a strong interaction between chitosan and olive oil since chitosan is hydrophobic in this form; subsequently, it is more prone to oily and lipophilic characteristics of olive oil (21, 22). On the other hand, if chitosan is applied with a higher degree of deacetylation, it turns to a hydrophilic form. Moreover, the presence of NH_2 _groups will cause a greater positive charge and a greater tendency to a negative charge in olive oil (23-25). Therefore, the above nanoemulsion will be formulated by loading the hydrophobic drug AmB in the oil phase of the emulsion coated with chitosan.

Due to the minimal therapeutic effects of dermal use of AmB, it was decided to apply this drug in emulsified form. Moreover, we selected more affordable surfactants, such as Span and Tween, with acceptable hydrophilic-lipophilic balance (HLB) (25). The HLB is a scale determining the hydrophilic-lipophilic balance of a surfactant that is characterized by calculating valence for various sections of the molecule, as explained by Griffin et al*.*)^26^(.

The HLB calculations for the NE and surfactant (O/W) were performed on 10-13, demonstrating a semi-transparent scattering NE for the temperature range of 8-18 °C and in this article (27-29) (Table 1). Based on the findings of the present research, there could be an interconnection between chitosan and triglycerides of olive oil. Two interactions exist between olive oil and chitosan (i.e., between the negative charge of olive oil and the positive charge of chitosan). 

The addition of AmB to the formulation resulted in the formulation of a new and more robust interaction between AmB and olive oil. Since both AmB and olive oil have a hydrophobic nature, they interact tightly via hydrophobe-hydrophobe interaction )^30^( and concentrate on the core of NE. As a result, chitosan left the emulsion toward the interface between two phases, ending in a more positively charged emulsion.

According to the obtained results, there could be interconnections between chitosan and olive oil triglycerides. The interaction between chitosan and olive oil depends on the degree of polymerization (DP) and the degree of deacetylation (DD) of chitosan. Chitosan solubility generally decreases with an increase in the degree of polymerization. Moreover, oil-conjunction capacity elevates with an increase in hydrophobicity of chitosan due to a reduced degree of deacetylation (50% < DD < 90%). Oil-conjunction capacity also increases with DD>90% due to electrostatic force between oil and chitosan (30).

Two interactions exist between the negative charge of olive oil and the positive charge of chitosan. The addition of AmB to the formulation resulted in the formation of a new and stronger interaction between AMB and olive oil. Since both AMB and olive oil have a hydrophobic nature, they interact tightly via hydrophobe-hydrophobe interaction and concentrate on the core of NE (31).

In comparison to the present study, Asthana S. *et al.* (32) developed a nanoemulsion (O/W) containing chitosan-coated AmB with the following differences. Firstly, in the mentioned study, soybean oil and soy lecithin were used to make nanoemulsions. Moreover, Tween 80 as a surfactant was applied for nanoemulsion stability and finally coated with low molecular weight chitosan with 75% and 85% acetylation degree. 

Secondly, Asthana S. et al*.* applied olive oil instead of seed oil and soybean lithium since it has a stronger interaction with chitosan. In addition to Twin 80, they also used Span 80 for more stability. Moreover, they applied chitosan with 85 and 75 degrees of deacetylation; nonetheless, in the present study, chitosan was used with low molecular weight and 90% acetylation degree. Drug release in the study by Asthana S. et al. was conducted at pH 7.5; nonetheless, in the present study, it was performed at pH 5.8, which is suitable for topical use. Thirdly, the average size of nanoemulsion in the referred study was reported to be between 100 and 150 nm, while this value in the current study was below 50. This size of nanoemulsion is more suitable for dermal absorption since a smaller size of nanoemulsion leads to better absorption. Fourthly, in the stated study, nanoemulsion showed a little toxicity for macrophages; nonetheless, no toxicity was observed in the current research (cc50 = 10.96 ± 0.11 g/m).

**Figure 4:**
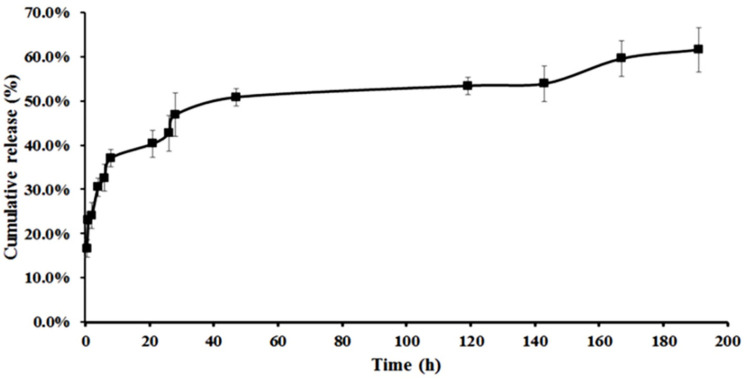
*In-vitro* drug release profiles of the CNE with AMB in pH 5.8

**Figure 5:**
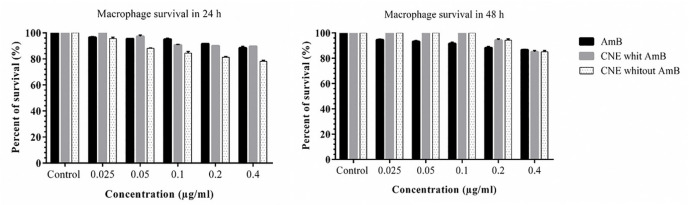
Changes in macrophage survival rate in 24 and 48 h with different concentrations AMB (control+), CNE with AMB or CNE without AMB three replicates per experiment

**Figure 6:**
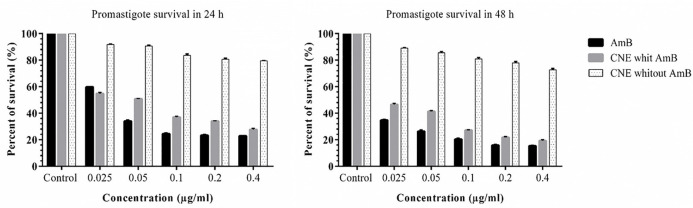
Changes in promastigote survival rate in 24 and 48 h with different concentrations AMB (control +), CNE with AMB or CNE without AMB three replicates per experiment

**Figure 7:**
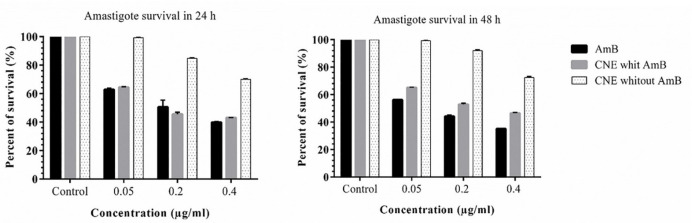
Changes in amastigote survival rate in 24 and 48 h with different concentrations AMB (control +), CNE with AMB and CNE without AMB three replicates per experiment

## Conclusion

In the present investigation, AmB was successfully infixed into the system by olive oil, surfactant, and followed by chitosan mixture with water. The main benefit of this method was mixing AmB with NE. Our data shows AmB- NE with or without chitosan was fixed in terms of particle size, pH, and AmB amount, after months. Also, no signs of clotting, sedimentation, and becoming two-phasic were observed after a long time. This study also demonstrated an improved integration of AmB in the skin transdermal system. Drug release in the emulsion formulations that were weak formerly improved when chitosan was added. Finally, the presence of chitosan in chitosan-coated nanoemulsions led to a better drug release both in the dialysis bag and in the culture medium compared to NE without chitosan and also led to the lack of carrier toxicity for macrophages.

## Ethics approval and consent to participate

Not applicable.

## Consent for publication

Not applicable.

## Availability of data and materials

Not applicable.

## Funding

This work was supported by the Department of Chemistry, Alborz Campus, University of Tehran, Tehran, Iran and Center for Research and Training in Skin Diseases and Leprosy, Tehran University of Medical Sciences, and Tehran, Iran.

## Conflict of interest statement

The authors have no conflicts of interest to declare.
